# Types of Insomnia Mediate the Relation between Anxiety and Depressive Symptoms Longitudinally in Late Life

**DOI:** 10.1093/geroni/igaa057.3318

**Published:** 2020-12-16

**Authors:** Courtney Bolstad, Michael Nadorff

**Affiliations:** Mississippi State University, Mississippi State, Mississippi, United States

## Abstract

The current study examined onset and maintenance insomnia as mediators of the relation between anxiety and depressive symptoms over a three-year period in a sample of older adults. We hypothesized that anxiety symptoms at timepoint one would significantly predict depressive symptoms at timepoint four, while controlling for depressive symptoms at timepoint one. We also hypothesized that this effect would be significantly reduced when adding onset and maintenance insomnia at timepoint two and three, respectively, as mediators. Participants included 3,484 older adults, ages 66 to 103 (M = 77, SD = 7), included in the National Health and Aging Trends Study who completed measures of types of insomnia, anxiety, and depressive symptoms at four different timepoints (2012 through 2015). The model showed a significant direct effect of anxiety on depressive symptoms at timepoint four, independent of baseline depressive symptoms. The relation was mediated by onset and maintenance insomnia, though a significant direct effect remained. Therefore, types of insomnia contribute to the development of depressive symptoms in older adults with anxiety symptoms over time, even when controlling for baseline depressive symptoms. Further, onset and maintenance insomnia are unique outcomes and predictors of anxiety and depressive symptoms, respectively, even when controlling for baseline depressive symptoms. Our findings provide a foundation for future intervention research with clinical samples that control for confounding variables in further elucidating the development and change in depressive symptoms among older adults with anxiety through onset and maintenance insomnia.

